# A Clinical Score to Identify Individuals at High Risk of Pancreatic Cancer in the General Population

**DOI:** 10.3390/diagnostics16152390

**Published:** 2026-07-29

**Authors:** Hyo Jeong Lee, Ji Seon Oh, Sehee Kim, Sung Won Park, Dongwook Oh, Tae Jun Song, Gwang-un Kim, Ji Young Choi, Jong Soo Lee, Hye-Sook Chang, Ji Young Lee

**Affiliations:** 1 Health Screening and Promotion Center, Asan Medical Center, University of Ulsan College of Medicine, 88 Olympic-ro 43-gil, Songpa-gu, Seoul 05505, Republic of Korea; 2 Department of Information Medicine, Asan Medical Center, University of Ulsan College of Medicine, Seoul 05505, Republic of Korea; 3 Department of Clinical Epidemiology and Biostatistics, Asan Medical Center, University of Ulsan College of Medicine, Seoul 05505, Republic of Korea; 4 Department of Gastroenterology, Asan Medical Center, University of Ulsan College of Medicine, Seoul 05505, Republic of Korea

**Keywords:** early detection of cancer, mass screening, pancreatic neoplasms, risk assessment

## Abstract

**Background/Objectives**: Early detection of pancreatic cancer (PC) remains challenging due to the lack of effective screening tools. We aimed to develop a clinically applicable model to identify individuals at high risk of PC using routine health check-up data. **Methods:** In a 1:4 matched case–control study (111 cases and 439 controls) using data collected between 2010 and 2018, PC cases were defined as individuals diagnosed with PC within 2 years of a health check-up. A model was developed using conditional logistic regression and validated in an independent cohort of 52,043 individuals (40 PC cases; 2019–2021). **Results:** Five risk factors were identified: carbohydrate antigen 19-9 ≥ 39 U/mL, hemoglobin A1c ≥ 6.5%, alkaline phosphatase > 110 IU/L, weight loss ≥ 5%, and dyspepsia. A 12-point risk scoring system was constructed, with an area under the curve of 0.784 in the development and 0.841 in the validation cohort. At a threshold of ≥5, the high-risk group had a significantly higher 2-year incidence of PC than the low-risk group (2.56% vs. 0.047%; *p* < 0.001), a 54.76-fold risk enrichment, with a negative predictive value of 99.95% and a number-needed-to-follow of 39. **Conclusions:** This simple, validated model effectively stratifies short-term PC risk using routine data, potentially facilitating targeted surveillance in the general population.

## 1. Introduction

Pancreatic cancer (PC) remains one of the most lethal malignancies worldwide, with a 5-year survival rate of only 13.9% [1]. Despite its relatively low incidence, PC ranks as the third leading cause of cancer-related death in the United States and the fourth in Europe [1,2]. The very poor prognosis is largely attributable to the fact that 80–85% of patients are diagnosed at an unresectable locally advanced or metastatic stage [3]; therefore, early detection is essential. However, a fundamental challenge in developing screening strategies for PC is its extremely low incidence, where the lifetime risk in the general population is approximately 1.7% [4]. Another major barrier is the scarcity of pre-diagnostic cohorts, as establishing large-scale pre-diagnostic cohorts requires substantial time, financial resources, and research infrastructure; most published biomarker candidates have been evaluated in samples from individuals previously diagnosed with PC [5].

The carbohydrate antigen 19-9 (CA 19-9) is the most widely used serum marker for PC and can be elevated up to 2 years before clinical diagnosis [6,7]. However, despite its relatively high sensitivity (79–81%) and specificity (82–90%) in symptomatic individuals, the positive predictive value of CA 19-9 remains extremely low (0.5–0.9%) due to the low incidence of PC, limiting its utility as a population-based screening tool [8]. In addition, the biological variability of CA 19-9 expression, including non-secretor status, and considerable tumor heterogeneity limit the utility of single biomarkers, reinforcing the requirement for multifactorial risk-prediction approaches [8,9,10,11]. Established risk factors for PC include older age, male sex, smoking, obesity, diabetes mellitus, chronic pancreatitis, and a family history of PC [12,13,14], while new-onset diabetes and unexplained weight loss have also emerged as potential early indicators of occult PC [15,16]. Current endeavors for early detection primarily focus on surveillance in high-risk groups, such as individuals with familial or genetic predisposition or those with premalignant pancreatic lesions [9,11]. Nevertheless, a substantial percentage of PC cases develop sporadically in the general population [17]. Therefore, identifying factors associated with an elevated risk of PC from routinely collected health check-up data could assist in stratifying individuals at high risk, who may subsequently benefit from targeted surveillance.

In this study, the aim was to develop a simple, reproducible, point-based risk scoring system to predict the 2-year risk of PC in the general Korean population, using routinely available laboratory findings and questionnaire-based clinical information.

## 2. Methods

### 2.1. Development of the Model

#### 2.1.1. Study Cohort and Case–Control Matching

The development dataset was derived from the anonymized Common Data Model (CDM) 3.0 of Asan Medical Center. It included retrospective cohort data from 4.95 million patients between May 1985 and December 2020. This database consists of de-identified, patient-level electronic health record (EHR) data, and health check-up data.

**Figure 1:**
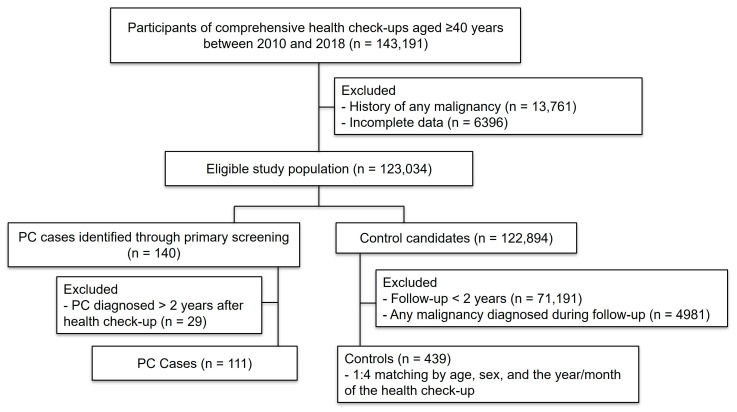
Flowchart of the study cohort.

To develop a prediction model for identifying individuals at high risk of PC, a 1:4 matched case–control study was conducted using CDM data. Initially, 143,191 participants aged ≥40 years who underwent comprehensive health check-ups between 2010 and 2018 were identified. After excluding individuals with a history of malignancy (*n* = 13,761) and those with incomplete data (*n* = 6396), a total of 123,034 participants were eligible. Cases of PC were defined as individuals newly diagnosed with PC using the International Classification of Diseases 10th revision codes, C25.x within 2 years of the health check-up (index date). Of the 140 initially identified patients, 27 were excluded because their diagnosis was >2 years after the check-up and two were excluded for endocrine pancreatic malignancy, yielding 111 PC cases. For the control group, individuals with <2 years of follow-up (*n* = 71,191) and those diagnosed with any malignancy during the follow-up period (*n* = 4981) were excluded from the 122,994 control candidates. From the remaining 46,822 eligible control candidates, 439 were matched to the 111 cases at a 4:1 ratio based on age, sex, and the year and month of the health check-up. The overall study flow is shown in Figure 1.

The study protocol was approved by the Institutional Review Board of Asan Medical Center (protocol no. 2024-0289; date of approval: 4 March 2024).

#### 2.1.2. Data Collection and Variables

The dataset included clinical characteristics obtained from a self-administered questionnaire, including family history of PC, smoking status, alcohol consumption, history of diabetes, ≥5% weight loss over the preceding year and gastrointestinal symptoms, anthropometric measurements, and laboratory test results. Laboratory variables included CA 19-9, carcinoembryonic antigen, hemoglobin A1c (HbA1c), aspartate aminotransferase (AST), alanine aminotransferase (ALT), alkaline phosphatase (ALP), gamma-glutamyl transferase (GGT), total bilirubin, total cholesterol, low-density lipoprotein cholesterol, triglycerides, white blood cell count, hemoglobin, high-sensitivity C-reactive protein (hsCRP), and albumin levels. Although the clinical cut-off for CA 19-9 is 37 IU/mL, the CA 19-9 cut-off was optimized at 39 IU/mL to ensure a specificity of ≥99% for PC, to develop the prediction model in the general population.

#### 2.1.3. Construction of the Risk Scoring System

Significant predictors were identified using univariable conditional logistic regression. To prevent multicollinearity, laboratory variables were separately evaluated before inclusion in the final multivariable model (Supplemental Table S1). A weighted risk scoring system was then developed based on the β-coefficients of the final model. Diagnostic performance metrics (sensitivity, specificity, and accuracy) were evaluated across score thresholds. Sensitivity was calculated as the proportion of PC cases correctly identified by the model, specificity as the proportion of controls who were correctly classified as negative, and accuracy as the proportion of all individuals who were correctly classified. The optimal threshold for identifying the “high-risk group” was set at the point within the development dataset that achieved ≥99% specificity.

### 2.2. Validation of the Model

For validation of the prediction model, an independent cohort of 52,413 individuals aged ≥ 40 years, who underwent health check-ups at the same institution between 2019 and 2021, were analyzed. Data were extracted from Asan Biomedical Research Environment, a clinical research data warehouse. After excluding individuals with history of any cancer (*n* = 333), missing data (*n* = 4), a history of pancreatic disease (*n* = 30), or PC diagnosed >2 years after the health check-up (*n* = 3), the final validation cohort consisted of 52,043 individuals. Within this cohort, 40 PC cases were diagnosed within 2 years after the check-up.

The pre-defined scoring system and high-risk threshold were applied to the validation cohort. Individuals were classified into high- and low-risk groups, and the 2-year incidence of PC was compared between the groups. Two-year incidence of PC was defined as the proportion of individuals diagnosed with PC within 2 years from the index date. Model performance was further quantified at the established threshold by assessing sensitivity, specificity, and accuracy.

### 2.3. Statistical Analysis

Continuous laboratory variables were categorized according to standard clinical reference ranges and are expressed as frequency (%). Categorical variables were compared using the Chi-square (χ^2^) or Fisher’s exact test. Model discrimination was evaluated by the area under the curve (AUC) from receiver operating characteristic curve analysis, with 95% confidence intervals (CIs). Overall model significance was evaluated using the likelihood ratio test (LRT) by comparing the final model with the null model, and additionally with a model containing CA 19-9 alone to assess the incremental contribution of the remaining predictors. Model calibration was assessed using a calibration plot with 1000 bootstrap repetitions, and the mean absolute error (MAE) was calculated to quantify the agreement between predicted and observed probabilities. Diagnostic performance at the pre-specified threshold was summarized as sensitivity, specificity, positive predictive value (PPV), and negative predictive value (NPV). Because PPV and NPV depend on disease prevalence, these were calculated only in the validation cohort, in which the observed 2-year incidence of PC was preserved, rather than in the matched case–control development dataset. The number-needed-to-follow (NNF), defined as the reciprocal of the PPV, was calculated to indicate the number of high-risk individuals requiring follow-up to identify one PC case within 2 years. Ninety-five percent confidence intervals for PPV and NPV were estimated using the Wilson score method, and the corresponding interval for NNF was derived from the reciprocals of the PPV interval limits. Age-related trends in the 2-year incidence of PC were evaluated using an ordinal trend test (*p* for trend). A two-sided *p* < 0.05 was considered to be statistically significant. All statistical analyses were performed using R software, version 4.4.2 (R Foundation for Statistical Computing, Vienna, Austria).

## 3. Results

### 3.1. Baseline Characteristics and Univariable Analysis

The matched case–control dataset included 111 patients with PC and 439 controls (Table 1). Among the 111 PC cases, 66.7% were male (*n* = 74). The age distribution of the PC cases was as follows: 40–49 years (6.3%), 50–59 years (36.9%), 60–69 years (33.3%), and ≥70 years (23.4%).

In univariable analyses, PC was associated with diabetes (odds ratio [OR], 2.70; 95% CI, 1.56–4.67), weight loss ≥ 5% over the preceding year (OR, 8.01; 95% CI, 4.14–15.47), and dyspepsia (OR, 1.97; 95% CI, 1.23–3.15). Among laboratory indices, a CA 19-9 level ≥ 39 U/mL had the strongest association with PC (OR, 27.58; 95% CI, 8.23–92.41). HbA1c ≥ 6.5%, elevated AST, ALT, ALP, GGT, bilirubin, and hsCRP levels, and anemia were also significantly associated with PC (all *p* < 0.01) (Table 1).

### 3.2. Development of High-Risk of PC Prediction Model

**Figure 2:**
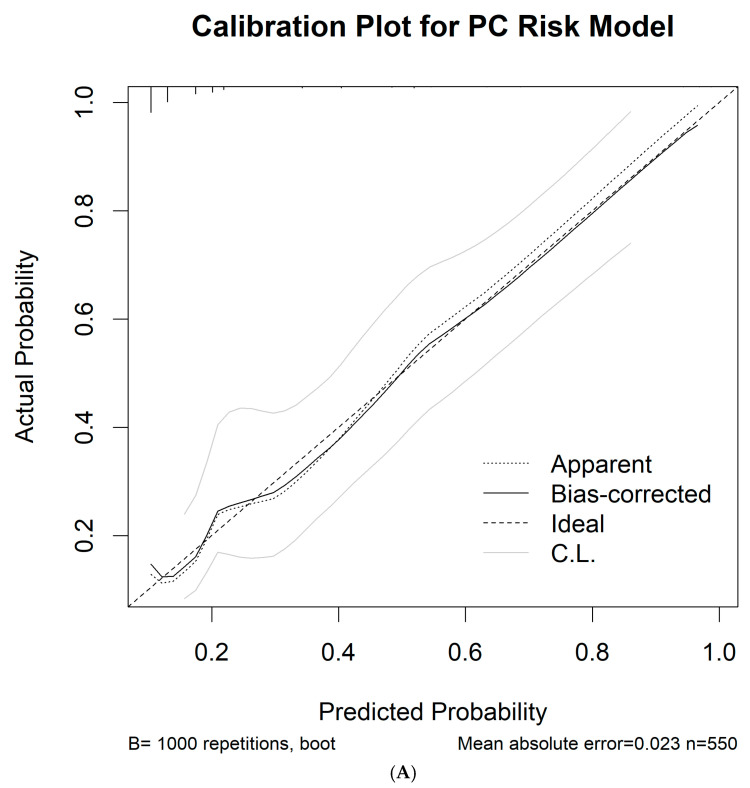

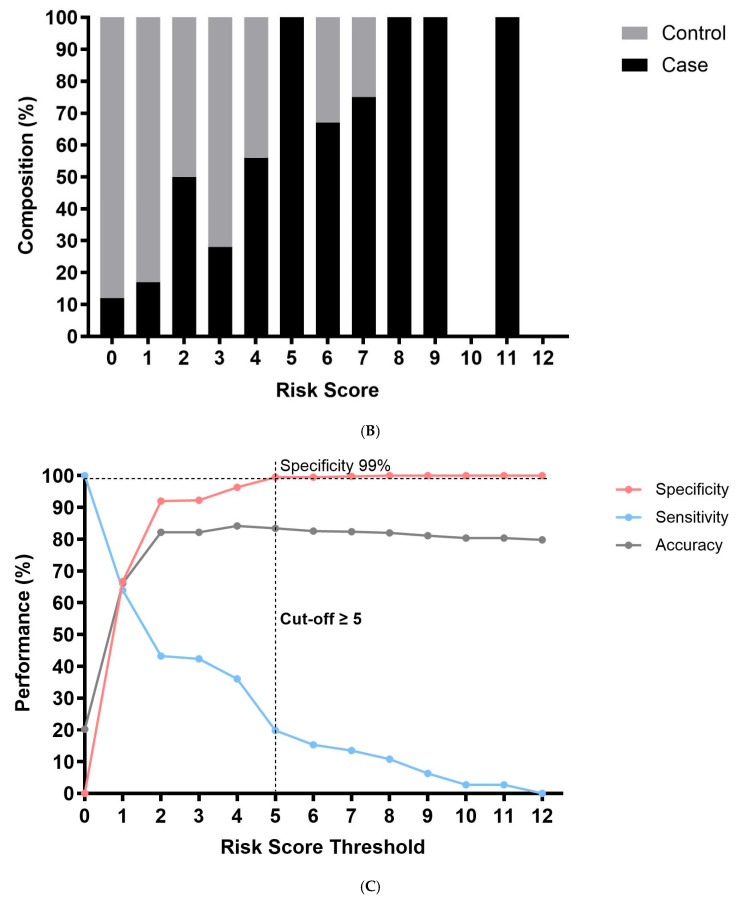
Calibration of the pancreatic cancer (PC) prediction model and evaluation of the derived risk scoring system in the development dataset. (**A**) Calibration plot of the PC risk prediction model. Dotted line, apparent performance; solid line, bias-corrected performance (1000 bootstrap repetitions, *n* = 550); dashed line, ideal calibration; gray lines, 95% confidence limits. Mean absolute error = 0.023. (**B**) Composition ratio of cases (Black) and controls (Gray) at each risk score. Each bar represents the relative proportion of cases and controls at each score, standardized to 100%. The proportion of cases increased progressively with higher risk scores. (**C**) Performance metrics (sensitivity, specificity, and accuracy) according to risk score thresholds. Specificity (Red), sensitivity (Blue), and accuracy (Gray) are plotted against increasing thresholds. A cut-off of ≥5 was selected as the optimal threshold, as it was the first point at which specificity reached ≥99%.

To identify predictors for the high-risk group for PC, clinical factors and laboratory indices were included. The clinical factors comprised weight loss ≥ 5% over the preceding year and dyspepsia, and laboratory indices included HbA1c ≥ 6.5%, ALP level > 110 IU/L, and anemia, based on multivariable analysis (Supplementary Table S1). Four multivariable conditional logistic regression models were constructed (Table 2). Model 2, which included clinical factors and laboratory variables excluding the CA 19-9 level, had moderate discrimination with an AUC of 0.748 (95% CI, 0.695–0.801). Adding the CA 19-9 level (Model 3) significantly improved performance (AUC, 0.785; 95% CI, 0.736–0.833; *p* < 0.001 compared with Model 2). Model 4 retained only five significant predictors from Model 3, namely, CA 19-9 level ≥ 39 U/mL, HbA1c ≥ 6.5%, ALP level > 110 IU/L, weight loss ≥ 5%, and dyspepsia, and comparable discrimination was maintained (AUC, 0.784; 95% CI, 0.735–0.833; *p* = 0.898 compared with Model 3). The LRT showed that Model 4 was statistically significant compared with the null model (χ^2^ = 94.48; degrees of freedom, 6; *p* < 0.001). Compared with a model containing CA 19-9 alone, the four remaining predictors jointly and significantly improved model fit (χ^2^ = 43.5; degrees of freedom = 5; *p* < 0.001), indicating that the score captures risk information independent of, and additive to, CA 19-9. The calibration plot of Model 4 showed good agreement between predicted and actual probabilities across the full range of risk, with an MAE of 0.023 (Figure 2A).

### 3.3. Performance of the Risk Scoring System in the Development Dataset

Using β-coefficients from Model 4, weighted risk scores were assigned to each predictor, yielding a total score range of 0 to 12 points (Table 2). In the development dataset, a clear dose–response relationship was observed, with the proportion of PC cases increasing at higher risk scores (Figure 2B). A score of ≥5 points was selected as the optimal threshold for defining the high-risk group, corresponding to a specificity ≥99% (Figure 2C and Supplemental Table S2). At this cut-off, the scoring system had a sensitivity of 19.82%, a specificity of 99.54%, and an accuracy of 83.45%.

### 3.4. Validation of Risk Scoring System in the Health Check-Up Cohort

**Figure 3:**
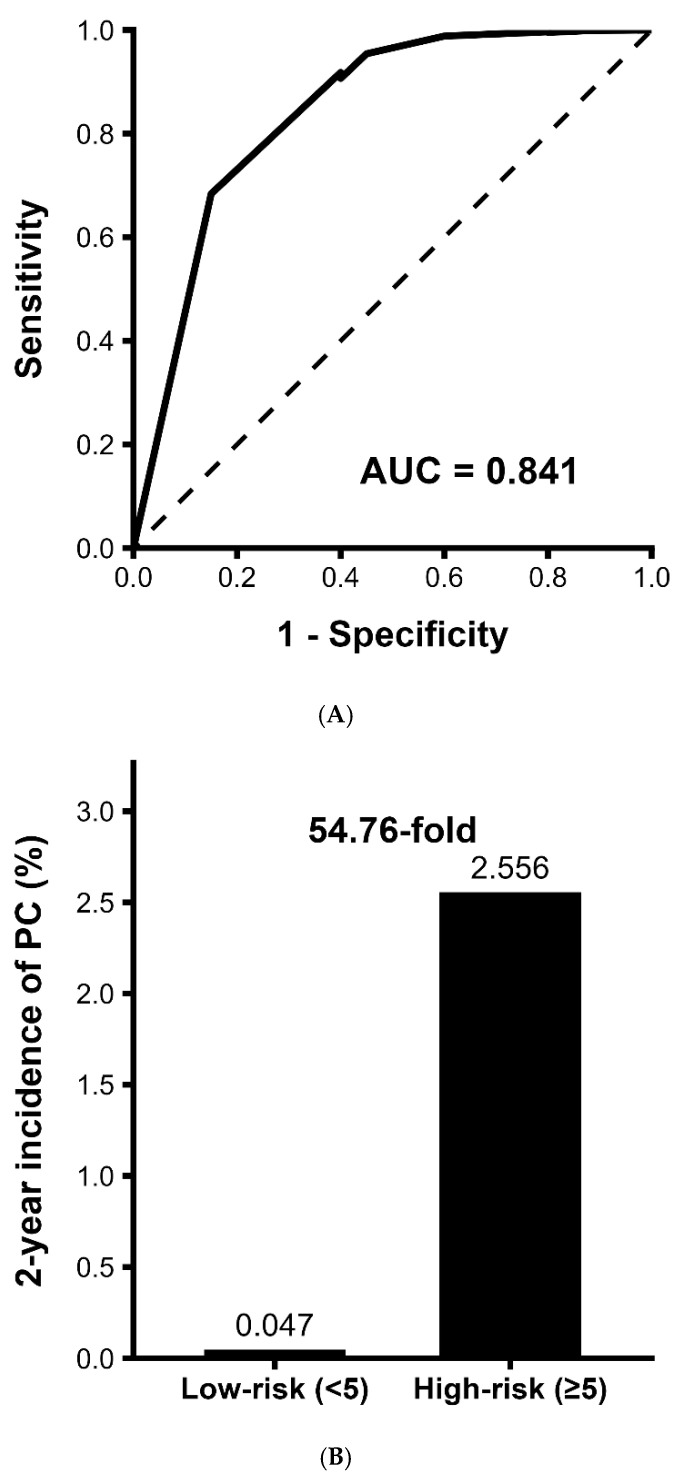
Discrimination and risk stratification of the Pancreatic Cancer Risk Scoring System in the health check-up validation cohort. (**A**) Receiver operating characteristic curve of the risk score. The model achieved an area under the curve (AUC) of 0.841 (95% confidence interval, 0.770–0.911). (**B**) Two-year incidence of pancreatic cancer (PC) according to the pre-defined risk score threshold (≥5). The 2-year incidence of PC was 0.047% in the low-risk group and 2.556% in the high-risk group, corresponding to a 54.76-fold higher incidence among individuals classified as high-risk (*p* < 0.001).

In 52,043 individuals undergoing health check-ups between 2019 and 2021, 40 (0.077%) developed PC within 2 years from the index date. Individuals who developed PC were older and had higher frequencies of diabetes, weight loss, dyspepsia, and elevated CA 19-9 and ALP levels, demonstrating that the clinical differences in the matched case–control dataset were consistently reproduced in the independent cohort (Supplemental Table S3). The model had an AUC of 0.841 (95% CI, 0.770–0.911) (Figure 3A), demonstrating good discriminative performance for predicting 2-year risk of developing PC. At the pre-defined threshold of ≥5 points, the model had a sensitivity of 40.00%, a specificity of 98.83%; the corresponding PPV and NPV were 2.56% (95% CI, 1.58–4.11%) and 99.95% (95% CI, 99.93–99.97%), respectively. Based on this threshold, 626 individuals (1.20% of the cohort) were classified as high-risk. The high-risk group had a significantly higher 2-year incidence of PC compared with the low-risk group (2.56% vs. 0.047%; *p* < 0.001), corresponding to a 54.76-fold difference (Figure 3B) and a NNF of 39 (95% CI, 24–63) to identify one PC case within 2 years.

Age-stratified analyses showed increasing absolute risk of PC with advancing age in the overall cohort and in the high-risk group (Table 3). Within the high-risk group, the 2-year incidence of PC was 0.43% in the 50–59-year group, 3.14% in the 60–69-year group, and 7.38% in the ≥70 years group, demonstrating a clear age-related increase in risk (*p* for trend < 0.001). Notably, for individuals aged ≥50 years, the high-risk group had a higher 2-year incidence of PC than the overall cohort across all age groups.

## 4. Discussion

A simple, point-based risk scoring system was developed and validated to predict 2-year risk of PC, using routine health check-up data from the general Korean population. In the high-risk group, the 2-year incidence of PC increased to 2.56%, compared with 0.047% in the low-risk group. Furthermore, the 2-year incidence of PC increased with age, affecting up to 7.38% of individuals aged ≥ 70 years. These findings suggest that routinely collected health check-up data can assist in identifying individuals at higher short-term risk of PC who may benefit from closer clinical surveillance.

Each of the five predictors included in the model has a plausible biological link to the pathophysiology of PC that are supported by the results of prior studies. The serum CA19-9 level is a clinically validated tumor marker in PC management, though its utility is limited by sub-optimal sensitivity in early stage disease [18] and false-negative results in individuals who are Lewis antigen-negative, with no or low secretion of CA 19-9 [19]. Despite these limitations, the CA19-9 level has demonstrated value in assessing tumor stage, resectability, overall survival, and therapeutic response [20]. In our cohort, most high-risk individuals who developed PC had an elevated CA 19-9 level, underscoring its central role in short-term risk detection. This is consistent with the previous finding that CA 19-9 levels begin to rise substantially as early as 2 years before diagnosis, with sensitivity reaching 50% at 99% specificity within 6 months of clinical presentation [21], suggesting a clinically meaningful window for risk stratification. Nevertheless, the score provided information beyond CA 19-9: adding the four remaining predictors to a CA 19-9-only model significantly improved model fit (LRT, χ^2^ = 43.5, df = 5, *p* < 0.001), and a model excluding CA 19-9 retained moderate discrimination (AUC, 0.748). Rather than replacing CA 19-9, the score integrates metabolic, hepatobiliary, and symptom-based signals to refine risk stratification—quantifying the degree of risk and identifying a small subset of individuals with a normal CA 19-9 level who nonetheless warranted closer attention.

Abnormal glucose metabolism is another important early signal of the development of PC [22] and new-onset diabetes has emerged as a potential predictor of early PC [15,16]. Unlike type 2 diabetes, new-onset diabetes in individuals with PC is paradoxically accompanied by progressive weight loss rather than weight gain. Although the pathogenesis remains to be fully elucidated, this phenomenon is postulated to reflect a paraneoplastic metabolic alteration driven by tumor-secreted factors that promote β-cell dysfunction and preferential subcutaneous fat lipolysis [22]. In our model, HbA1c ≥ 6.5% and weight loss ≥ 5% were retained as independent predictors, consistent with these established pathophysiological mechanisms. An elevated ALP level may be relevant in early PC, potentially reflecting subtle biliary obstruction or hepatobiliary involvement before overt jaundice develops [23]. Finally, although dyspepsia is individually non-specific, this upper gastrointestinal symptom is more frequently present in the months preceding diagnosis of PC [24,25]. The inclusion of dyspepsia in the model reflects its potential value as a clinical warning sign when considered in the context of concurrent metabolic and biochemical abnormalities. Collectively, the five predictors in the model reflect complementary clinical, metabolic, and hepatobiliary pathways through which PC can manifest, before overt diagnosis, which may assist in explaining the improved discriminative performance when they are combined in a composite score.

Several prediction models have been developed to estimate the short-term risk of developing PC using clinical data [15,16,26,27]. The PC risk prediction model (Prism) derived from EHR data across 55 US healthcare organizations, achieved AUCs of 0.826 (neural network) and 0.800 (logistic regression) in the general population for the prediction of the risk of developing pancreatic adenocarcinoma, 6–18 months before diagnosis [27]. Other models, including the Boursi model [15] and the Enriching New-Onset Diabetes for Pancreatic Cancer (ENDPAC) score [16], have focused on identifying the risk of PC, specifically among patients with new-onset diabetes, incorporating changes in weight, blood glucose, and metabolic parameters. While these approaches have demonstrated promising discrimination, they require comprehensive EHR infrastructure with large-scale longitudinal data or are applicable only to a restricted subgroup of patients with new-onset diabetes. By contrast, our model identifies high-risk individuals using only five variables that are routinely collected during standard health check-ups, regardless of diabetes status, without the requirement for complex computational infrastructure or institutional data integration. Given that a substantial proportion of PC cases develop sporadically in individuals without diabetes or established risk factors, risk stratification tools applicable to the broader population are critically required. Our model, developed and validated in a general health check-up cohort, addresses this unmet need, and it may assist primary care providers in routine clinical practice to efficiently identify candidates for further diagnostic evaluation. In the validation cohort, the model achieved a 54.76-fold enrichment of the incidence of PC in the high-risk group while limiting the screening burden to only 1.2% of the total population. Additionally, age-stratified analysis revealed that the 2-year incidence of PC, in high-risk individuals aged ≥ 70 years, reached 7.38%, supporting the rationale for further studies of individuals in this group.

This study has several strengths including the use of pre-diagnostic data from large, health check-up data, and validation in an independent cohort. The model targets short-term (2-year) risk, aligning with the clinical course of PC and increasing its relevance for early detection. Notably, this high-risk group was identified using only routinely available clinical and laboratory data, without genetic testing; therefore, the point-based structure can meaningfully stratify the risk of PC in the general population, enhance interpretability, and facilitate clinical adoption.

However, several limitations are noted. First, the model was developed and validated within a single center and a homogeneous ethnic population, which may limit its generalizability. Second, the absolute number of PC cases was small, which may limit the precision of risk estimates. Third, the development dataset was based on a matched case–control design, which does not directly reflect the true prevalence of PC in the general population. Although validation was performed in an independent cohort to address this issue, the case–control framework may have influenced the estimated magnitude of associations. Finally, some important risk factors, including genetic susceptibility, presence of pancreatic cysts, and detailed medication history, were not included. Future studies should prioritize external validation across diverse populations and assessment of clinical utility in prospective cohorts. Analyses incorporating longitudinal biomarker trends or broader clinical factors using interpretable machine-learning approaches may also be informative. Prospective surveillance studies in individuals classified as high-risk are essential to determine whether risk-based follow-up leads to earlier detection or improved outcomes.

In conclusion, we developed and validated a simple point-based model to estimate the 2-year risk of PC using routinely collected health check-up data. The score identified a small subgroup within the general population with substantially elevated short-term risk while maintaining high specificity, suggesting a potential value in identifying individuals who may benefit from closer clinical follow-up. Prospective studies are required to determine whether applying this score in routine health check-ups can facilitate earlier detection and enhance clinical decision-making.

## Figures and Tables

**Table 1:** Baseline characteristics and univariable conditional logistic regression analysis for the risk of developing pancreatic cancer.

Variable	PC (*n* = 111)	Control (*n* = 439)	OR (95% CI)	*p*
Sex			Matched	
Female	37 (33.3%)	143 (32.6%)		
Male	74 (66.7%)	296 (67.4%)		
Age, years			Matched	
40–49	7 (6.3%)	28 (6.4%)		
50–59	41 (36.9%)	164 (37.3%)		
60–69	37 (33.3%)	148 (33.7%)		
≥70	26 (23.4%)	99 (22.5%)		
Body mass index, kg/m^2^				
18.5–22.9	42 (37.8%)	167 (38.0%)	Ref.	
<18.5	4 (3.6%)	10 (2.3%)	1.52 (0.45–5.12)	0.497
23–24.9	27 (24.3%)	125 (28.5%)	0.87 (0.51–1.48)	0.605
≥25	38 (34.2%)	137 (31.2%)	1.10 (0.67–1.80)	0.713
Smoking history, pack-years				
>10	44 (39.6%)	200 (45.6%)	0.70 (0.42–1.19)	0.189
≤10	67 (60.4%)	239 (54.4%)	Ref.	
Alcohol, drinks/week				
≥4	6 (5.4%)	40 (9.1%)	0.57 (0.23–1.39)	0.217
<4	105 (94.6%)	399 (90.9%)	Ref.	
Diabetes mellitus				
Yes	33 (29.7%)	71 (16.2%)	2.70 (1.56–4.67)	<0.001
No	78 (70.3%)	368 (83.8%)	Ref.	
Family history of PC				
Yes	3 (2.7%)	19 (4.3%)	0.63 (0.18–2.14)	0.454
No	108 (97.3%)	420 (95.7%)	Ref.	
Weight loss ≥ 5% *				
Yes	30 (27.0%)	20 (4.6%)	8.01 (4.14–15.47)	<0.001
No	81 (73.0%)	419 (95.4%)	Ref.	
Bloating				
Yes	21 (18.9%)	64 (14.6%)	1.40 (0.80–2.42)	0.235
No	90 (81.1%)	375 (85.4%)	Ref.	
Dyspepsia				
Yes	34 (30.6%)	79 (18.0%)	1.97 (1.23–3.15)	0.005
No	77 (69.4%)	360 (82.0%)	Ref.	
CA 19-9				
≥39 U/mL	22 (19.8%)	4 (0.9%)	27.58 (8.23–92.41)	<0.001
<39 U/mL	89 (80.2%)	435 (99.1%)	Ref.	
CEA				
≥5 ng/mL	2 (1.8%)	4 (0.9%)	2.00 (0.37–10.92)	0.423
<5 ng/mL	109 (98.2%)	435 (99.1%)	Ref.	
HbA1c, %				
<5.7	46 (41.4%)	253 (57.6%)	Ref.	
5.7–6.4	37 (33.3%)	138 (31.4%)	1.46 (0.90–2.39)	0.128
≥6.5	28 (25.2%)	48 (10.9%)	3.37 (1.87–6.07)	<0.001
AST				
>40 IU/L	19 (17.1%)	36 (8.2%)	2.95 (1.54–5.64)	0.001
≤40 IU/L	92 (82.9%)	403 (91.8%)	Ref.	
ALT				
>40 IU/L	21 (18.9%)	33 (7.5%)	2.37 (1.29–4.36)	0.006
≤40 IU/L	90 (81.1%)	406 (92.5%)	Ref.	
ALP				
>110 IU/L	15 (13.5%)	11 (2.5%)	5.80 (2.60–12.95)	<0.001
≤110 IU/L	96 (86.5%)	428 (97.5%)	Ref.	
GGT (>36 IU/L in females, >60 IU/L in males)				
Elevated	23 (20.7%)	40 (9.1%)	2.71 (1.52–4.83)	<0.001
Normal	88 (79.3%)	399 (90.9%)	Ref.	
Total bilirubin				
>1.2 mg/dL	18 (16.2%)	64 (14.6%)	1.15 (0.65–2.05)	0.629
≤1.2 mg/dL	93 (83.8%)	375 (85.4%)	Ref.	
Total cholesterol				
>199 mg/dL	32 (28.8%)	158 (36.0%)	0.70 (0.44–1.12)	0.140
≤199 mg/dL	79 (71.2%)	281 (64.0%)	Ref.	
LDL-C				
>129 mg/dL	33 (29.7%)	163 (37.1%)	0.70 (0.44–1.11)	0.132
≤129 mg/dL	78 (70.3%)	276 (62.9%)	Ref.	
Triglyceride				
≥150 mg/dL	20 (18.0%)	90 (20.5%)	0.86 (0.50–1.48)	0.588
<150 mg/dL	91 (82.0%)	349 (79.5%)	Ref.	
WBC count				
≥10,000/µL	3 (2.7%)	8 (1.8%)	1.50 (0.40–5.65)	0.549
<10,000/µL	108 (97.3%)	431 (98.2%)	Ref.	
Hemoglobin (<12 g/dL in females, <13 g/dL in males)				
Low (anemia)	15 (13.5%)	21 (4.8%)	3.13 (1.50–6.54)	0.002
Normal	96 (86.5%)	418 (95.2%)	Ref.	
hsCRP				
>0.6 mg/dL	10 (9.0%)	11 (2.5%)	4.02 (1.55–10.39)	0.004
≤0.6 mg/dL	101 (91.0%)	428 (97.5%)	Ref.	
Albumin				
<3.5 g/dL	4 (3.6%)	7 (1.6%)	2.37 (0.62–9.10)	0.208
≥3.5 g/dL	107 (96.4%)	432 (98.4%)	Ref.	

Data are presented as number (%). ALT, alanine aminotransferase; ALP, alkaline phosphatase; AST, aspartate aminotransferase; CA 19-9, carbohydrate antigen 19-9; CEA, carcinoembryonic antigen; CI, confidence interval; GGT, gamma-glutamyl transferase; HbA1c, hemoglobin A1c; hsCRP, high-sensitivity C-reactive protein; LDL-C, low density lipoprotein cholesterol; OR, odds ratio; PC, pancreatic cancer; WBC, white blood cell count. * Weight loss was defined as a ≥5% reduction in body weight over the preceding year.

**Table 2:** Multivariable models for the risk of developing pancreatic cancer and assigned risk scores.

	Model 1			Model 2			Model 3			Model 4				
Variables	OR	95% CI	*p*	OR	95% CI	*p*	OR	95% CI	*p*	OR	95% CI	*p*	β	Assigned Score
CA 19-9 ≥39 U/mL	27.58	8.23–92.41	<0.001				13.25	3.54–49.55	<0.001	15.12	4.01–56.98	<0.001	2.716	4
HbA1c, %														
<5.7				Ref.			Ref.			Ref.				
5.7–6.4				1.37	0.80–2.35	0.253	1.20	0.68–2.11	0.530	1.22	0.70–2.14	0.487	0.199	
6.5				2.74	1.36–5.51	0.005	2.21	1.05–4.67	0.037	2.26	1.08–4.75	0.031	0.817	1
ALP >110 IU/L				5.58	2.22–14.02	<0.001	5.15	1.87–14.15	0.001	5.52	2.05–14.84	<0.001	1.708	3
Anemia *				2.43	1.04–5.66	0.040	1.99	0.80–4.91	0.137					
Weight loss ^†^				6.21	3.01–12.81	<0.001	5.35	2.45–11.69	<0.001	5.57	2.56–12.10	<0.001	1.717	3
Dyspepsia				2.21	1.26–3.86	0.006	1.86	1.03–3.37	0.040	1.87	1.04–3.37	0.038	0.625	1

ALP, alkaline phosphatase; CA 19-9, carbohydrate antigen 19-9; CI, confidence interval; HbA1c, hemoglobin A1c; OR, odds ratio; β, regression coefficients. * Anemia was defined as hemoglobin < 12 g/dL in females and <13 g/dL in males. ^†^ Weight loss was defined as a ≥5% reduction in body weight over the preceding year.

**Table 3:** Application of the Pancreatic Cancer Risk Scoring System by age group in the health check-up validation cohort.

Age (Years)	Total Cohort			High-Risk Group (Score ≥ 5)		
	Number, n	PC, n	Two-Year Incidence	Number, n	PC, n	Two-Year Incidence
40–49	13,753	0	0.00%	81	0	0.00%
50–59	21,635	9	0.04%	232	1	0.43%
60–69	12,505	16	0.13%	191	6	3.14%
≥70	4150	15	0.36%	122	9	7.38%
Total	52,043	40	0.08%	626	16	2.56%

PC, pancreatic cancer.

## Data Availability

Data available on request due to restrictions: The data presented in this study are available on request from the corresponding author due to restrictions imposed by the Institutional Review Board of Asan Medical Center and institutional data governance policies.

## Supplementary Materials

The following supporting information can be downloaded at: https://www.mdpi.com/article/10.3390/diagnostics16152390/s1, Supplemental Table S1. Multivariable conditional logistic regression analysis of laboratory variables in the development dataset. Supplemental Table S2. Performance metrics according to risk score thresholds in the development dataset. Supplemental Table S3. Baseline characteristics of the health check-up validation cohort.
