# Supplementary material for: A Clinical Score to Identify Individuals at High Risk of Pancreatic Cancer in the General Population
Source: Diagnostics (Basel). 2026 Jul 29;16(15):2390. doi: 10.3390/diagnostics16152390 (PMC13463860; doi:10.3390/diagnostics16152390)
Supplement: Supplementary file 1 [file diagnostics-16-02390-s001.zip › diagnostics-4468066-supplementary.pdf]

Supplemental Materials

Supplemental Table S1. Multivariable conditional logistic regression analysis of laboratory variables in the development dataset.

**Variables**	**OR**	**95% CI**	**p**
**HbA1c, %**			
**<5.7**	Ref.		
**5.7-6.4**	1.33	0.79 - 2.24	0.285
**≥6.5**	2.86	1.52 - 5.40	0.001
**AST >40 IU/L**	0.95	0.37 - 2.40	0.909
**ALT >40 IU/L**	1.40	0.58 - 3.41	0.464
**ALP >110 IU/L**	3.94	1.58 - 9.84	0.003
**GGT >36 IU/L in females, >60 IU/L in males**	1.54	0.74 - 3.22	0.248
**Hemoglobin <12 g/dL in females, <13 g/dL in males**	2.47	1.12 - 5.47	0.025
**hsCRP >0.6 mg/dL**	2.66	0.96 - 7.35	0.059

ALP, alkaline phosphatase; ALT, alanine aminotransferase; AST, aspartate aminotransferase; CI, confidence interval; GGT, gamma-glutamyl transferase; HbA1c, Hemoglobin A1c; hsCRP, high-sensitivity C-reactive protein; OR, Odds ratio.

Supplemental Table S2. Performance metrics according to risk score thresholds in the development dataset.

**Score Thresholds**	**Case**	**Control**	**Sensitivity**	**Specificity**	**Accuracy**
**≥0**	111	439	100.00%	0.00%	20.18%
**≥1**	71	146	63.96%	66.74%	66.18%
**≥2**	48	35	43.24%	92.03%	82.18%
**≥3**	47	34	42.34%	92.26%	82.18%
**≥4**	40	16	36.04%	96.36%	84.18%
**≥5**	22	2	19.82%	99.54%	83.45%
**≥6**	17	2	15.32%	99.54%	82.55%
**≥7**	15	1	13.51%	99.77%	82.36%
**≥8**	12	0	10.81%	100.00%	82.00%
**≥9**	7	0	6.31%	100.00%	81.09%
**≥10**	3	0	2.70%	100.00%	80.36%
**≥11**	3	0	2.70%	100.00%	80.36%
**12**	0	0	0.00%	100.00%	79.82%

PC, pancreatic cancer

Supplemental Table S3. Baseline characteristics of the health check-up validation cohort

Variables	PC within 2 years (n=40)	No PC (n =52,003)	p
**Sex**			0.4
**Female**	16 (40.0%)	23,977 (46.1%)	
**Male**	24 (60.0%)	28,026 (53.9%)	
**Age, years**			<0.001
**40-49**	0 (0.0%)	13,753 (26.4%)	
**50-59**	9 (22.5%)	21,626 (41.6%)	
**60-69**	16 (40.0%)	12,489 (24.0%)	
**≥70**	15 (37.5%)	4,135 (8.0%)	
**Diabetes**	15 (37.5%)	5,095 (9.8%)	<0.001
**Weight loss ≥5%***	7 (17.5%)	1,566 (3.0%)	<0.001
**Dyspepsia**	13 (32.5%)	9,965 (19.2%)	0.032
**CA 19-9 ≥39 U/mL**	19 (47.5%)	1,128 (2.2%)	<0.001
**HbA1c, %**			<0.001
**<5.7**	10 (25.0%)	32,655 (62.8%)	
**5.7-6.4**	15 (37.5%)	14,840 (28.5%)	
**≥6.5**	15 (37.5%)	4,508 (8.7%)	
**ALP >110 IU/L**	7 (17.5%)	1,799 (3.5%)	<0.001

Data are presented as number (%).

ALP, alkaline phosphatase; CA 19-9, Carbohydrate Antigen 19-9; HbA1c, Hemoglobin A1c; PC, pancreatic cancer.

*Weight loss was defined as a ≥5% reduction in body weight over the preceding year.
